# 5,5′-Di-4-pyridyl-2,2′-(5-*tert*-butyl-*m*-phenyl­ene)bis­(1,3,4-oxadiazole)

**DOI:** 10.1107/S1600536809027056

**Published:** 2009-07-15

**Authors:** Katsuhiko Ono, Kenichi Tsukamoto, Masaaki Tomura

**Affiliations:** a Department of Materials Science and Engineering, Nagoya Institute of Technology, Gokiso, Showa-ku, Nagoya 466-8555, Japan; b Institute for Molecular Science, Myodaiji, Okazaki 444-8585, Japan

## Abstract

The title compound, C_24_H_20_N_6_O_2_, is a novel 1,3,4-oxadiazole derivative which has potential as an electron-transporting material in organic electroluminescent (EL) devices. In the crystal, the mol­ecular framework is almost planar with an r.m.s. deviation of 0.091 (4) Å and it exists in an *E* form. Intra­molecular C—H⋯O and C—H⋯N hydrogen bonds are observed between the benzene and 1,3,4-oxadiazole rings. The *tert*-butyl group is disordered over two sites, with occupancy factors of 0.78 (1) and 0.22 (1) for the major and minor orientations, respectively. In the crystal structure, mol­ecules aggregate *via* C—H⋯N inter­actions, forming mol­ecular tapes along the *b* axis, which aggregate to form a mol­ecular sheet *via* C—H⋯N inter­actions.

## Related literature

The application of 1,3,4-oxadiazole derivatives as electron-transporting materials in EL devices has been reported by Hughes & Bryce (2005 ▶). For related structures, including the 1,3,4-oxadiazole system, see: Ono *et al.*(2005 ▶, 2008 ▶).
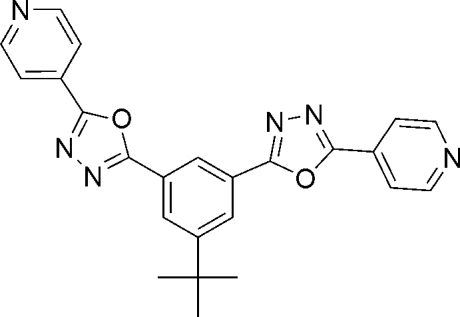


## Experimental

### 

#### Crystal data

C_24_H_20_N_6_O_2_
*M* *_r_* = 424.46
Monoclinic, 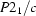

*a* = 5.8778 (10) Å
*b* = 14.767 (3) Å
*c* = 25.298 (6) Å
β = 90.635 (10)°
*V* = 2195.7 (8) Å^3^
*Z* = 4
Mo *K*α radiation
μ = 0.09 mm^−1^
*T* = 296 K
0.25 × 0.13 × 0.10 mm

#### Data collection

Rigaku Mercury CCD diffractometer
Absorption correction: none
16667 measured reflections
4956 independent reflections
1548 reflections with *I* > 2σ(*I*)
*R*_int_ = 0.112

#### Refinement

*R*[*F* ^2^ > 2σ(*F* ^2^)] = 0.070
*wR*(*F* ^2^) = 0.172
*S* = 0.90
4956 reflections
323 parameters
16 restraints
H-atom parameters constrained
Δρ_max_ = 0.23 e Å^−3^
Δρ_min_ = −0.13 e Å^−3^

### 

Data collection: *CrystalClear* (Rigaku/MSC, 2006 ▶); cell refinement: *CrystalClear*; data reduction: *TEXSAN* (Rigaku/MSC, 2004 ▶); program(s) used to solve structure: *SHELXS97* (Sheldrick, 2008 ▶); program(s) used to refine structure: *SHELXL97* (Sheldrick, 2008 ▶); molecular graphics: *PLATON* (Spek, 2009 ▶) and *Mercury* (Macrae *et al.*, 2006 ▶); software used to prepare material for publication: *SHELXL97*.

## Supplementary Material

Crystal structure: contains datablocks I, global. DOI: 10.1107/S1600536809027056/rn2057sup1.cif

Structure factors: contains datablocks I. DOI: 10.1107/S1600536809027056/rn2057Isup2.hkl

Additional supplementary materials:  crystallographic information; 3D view; checkCIF report

## Figures and Tables

**Table 1:** Hydrogen-bond geometry (Å, °)

*D*—H⋯*A*	*D*—H	H⋯*A*	*D*⋯*A*	*D*—H⋯*A*
C2—H2⋯O1	0.93	2.54	2.860 (4)	101
C6—H6⋯N4	0.93	2.61	2.928 (5)	100
C11—H11⋯N2^i^	0.93	2.50	3.406 (6)	164
C17—H17⋯N5^ii^	0.93	2.56	3.430 (5)	156

Symmetry codes: (i) 
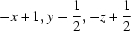
; (ii) 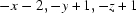.

**Fig. 1.**
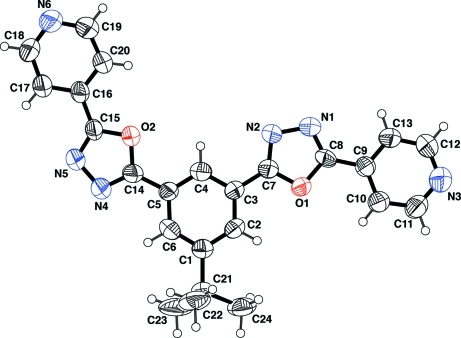
The molecular structure of (I), with atom labels and 50% probability displacement ellipsoids for non-H atoms and H atoms are shown as small spheres of arbitrary radii. The disordered atoms (C25–C27) of the tert-butyl group are omitted for clarity.

**Fig. 2.**
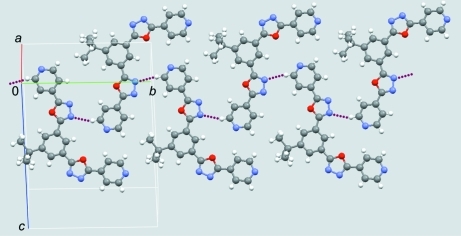
Partial diagram of (I), showing a molecular tape along the b axis.

**Fig. 3.**
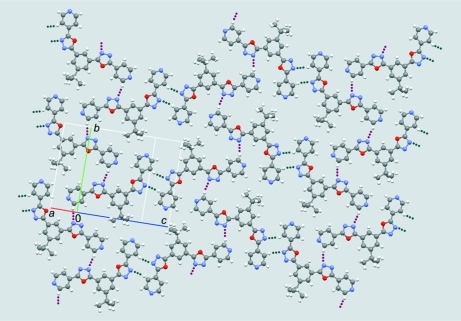
The packing diagram of (I), showing a molecular sheet.

## supplementary crystallographic information

### Comment

1,3,4-Oxadiazole derivatives are highly attractive compounds in the research and development of materials for organic electroluminescent (EL) devices since these compounds possess high electron-accepting properties and exhibit strong fluorescence with high quantum yields. Up to now, various electron-transporting materials with 1,3,4-oxadiazole system have been synthesized and investigated for application in EL devices (Hughes & Bryce, 2005). The research of crystal structures is important for the design of electron-transporting materials (Ono *et al.* 2005; Ono *et al.* 2008), because these properties depend on their molecular arrangements in the solid state. Thus, we synthesized the title compound (I) as a novel 1,3,4-oxadiazole derivative and investigated its molecular and crystal structure.

The molecular structure of (I) is shown in Fig. 1. The molecular framework is almost planar with an r.m.s. deviation of 0.091 (4) Å and exists in an *E*–form. Intramolecular C—H···O [2.860 (4) Å for C···O] and C—H···N [2.928 (5) Å for C···N] hydrogen bonds are observed between the benzene and 1,3,4-oxadiazole rings. The *tert*-butyl group is disordered over two sites with occupancy factors of 0.78 (1) and 0.22 (1) for the major and minor orientations, respectively. The molecular structure is characterized by molecular tapes along the *b* axis formed by C—H···N interactions [3.406 (6) Å for C···N], as shown in Fig. 2. The molecular tapes form stacks, where the distance between the molecular planes is 3.30 Å. The molecular tapes also aggregate to form a molecular sheet *via* C—H···N interactions [3.430 (5) Å for C···N] (Fig. 3). The title compound with the sheet-type network and stacking arrangement has potential as an electron-transporting material in EL devices.

### Experimental

The synthesis of the title compound (I) consists of two reaction steps as follows: A mixture of 5-*tert*-butylisophthalic dihydrazide (2.50 g, 10.0 mmol) and isonicotinoyl chloride hydrochloride (3.92 g, 22.0 mmol) in dry pyridine (200 ml) was stirred for 40 min at 0 °C under nitrogen. Then, the reaction mixture was refluxed for 5 h. After removal of the solvent, cold water was added. The white precipitate was filtered and washed with cold water and ether to afford compound (II) (3.53 g, 77%) as a white solid, which was used for the following reaction. A mixture of compound (II) (0.40 g, 0.87 mmol) and polyphosphoric acid (PPA) (40 g) was stirred at 180 °C for 2 h. After cooling, the reaction mixture was poured into water. The aqueous solution was basified to pH 9 with an aqueous NaOH solution, and dichloromethane was added. The organic layer was separated and the aqueous layer was extracted twice with dichloromethane. The combined organic solution was dried over Na_2_SO_4_ and concentrated. The residue was separated by column chromatography on alumina gel to afford the title compound (0.26 g, 71%) as a white powder. Colorless crystals of the compound, suitable for X-ray analysis were grown from a solution of CHCl_3_ and hexane.

### Refinement

All H atoms were placed in geometrically calculated positions, with C—H = 0.93 (aromatic) and 0.96 (methyl) Å and *U*_iso_(H) = 1.2*U*_eq_(C) (aromatic) and 1.5*U*_eq_(C) (methyl), and refined using a riding model.

### Crystal data

**Table:**

C_24_H_20_N_6_O_2_	*F*(000) = 888
*M**_r_* = 424.46	*D*_x_ = 1.284 Mg m^−^^3^
Monoclinic, *P*2_1_/*c*	Melting point: 529 K
Hall symbol: -P 2ybc	Mo *K*α radiation, λ = 0.71070 Å
*a* = 5.8778 (10) Å	Cell parameters from 1816 reflections
*b* = 14.767 (3) Å	θ = 3.2–27.5°
*c* = 25.298 (6) Å	µ = 0.09 mm^−^^1^
β = 90.635 (10)°	*T* = 296 K
*V* = 2195.7 (8) Å^3^	Prism, yellow
*Z* = 4	0.25 × 0.13 × 0.10 mm

### Data collection

**Table:**

Rigaku Mercury CCD diffractometer	1548 reflections with *I* > 2σ(*I*)
Radiation source: rotating anode	*R*_int_ = 0.112
graphite monochromator	θ_max_ = 27.5°, θ_min_ = 3.2°
Detector resolution: 14.62 pixels mm^-1^	*h* = −5→7
φ and ω scans	*k* = −19→14
16667 measured reflections	*l* = −32→22
4956 independent reflections	

### Refinement

**Table:**

Refinement on *F*^2^	Primary atom site location: structure-invariant direct methods
Least-squares matrix: full	Secondary atom site location: difference Fourier map
*R*[*F*^2^ > 2σ(*F*^2^)] = 0.070	Hydrogen site location: inferred from neighbouring sites
*wR*(*F*^2^) = 0.172	H-atom parameters constrained
*S* = 0.90	*w* = 1/[σ^2^(*F*_o_^2^) + (0.0474*P*)^2^] where *P* = (*F*_o_^2^ + 2*F*_c_^2^)/3
4956 reflections	(Δ/σ)_max_ = 0.004
323 parameters	Δρ_max_ = 0.23 e Å^−^^3^
16 restraints	Δρ_min_ = −0.13 e Å^−^^3^

### Special details

**Table:**

Experimental. ^1^H NMR (DMSO-d_6_, δ p.p.m.): 1.46 (s, 9H), 8.15 (d, J = 5.5 Hz, 4H), 8.36 (s, 2H), 8.61 (s, 1H), 8.88 (d, J = 5.5 Hz, 4H); MS (EI): m/z 424 (M^+^), 409.
Geometry. All e.s.d.'s (except the e.s.d. in the dihedral angle between two l.s. planes) are estimated using the full covariance matrix. The cell e.s.d.'s are taken into account individually in the estimation of e.s.d.'s in distances, angles and torsion angles; correlations between e.s.d.'s in cell parameters are only used when they are defined by crystal symmetry. An approximate (isotropic) treatment of cell e.s.d.'s is used for estimating e.s.d.'s involving l.s. planes.
Refinement. Three methyl groups of the *tert*-butyl group are disordered over two sites (C22–C24 and C25–C27) with occupancies of 0.78 (1):0.22 (1). The values were determined by refining site occupancies.Refinement of *F*^2^ against ALL reflections. The weighted *R*-factor *wR* and goodness of fit *S* are based on *F*^2^, conventional *R*-factors *R* are based on *F*, with *F* set to zero for negative *F*^2^. The threshold expression of *F*^2^ > σ(*F*^2^) is used only for calculating *R*-factors(gt) *etc*. and is not relevant to the choice of reflections for refinement. *R*-factors based on *F*^2^ are statistically about twice as large as those based on *F*, and *R*- factors based on ALL data will be even larger.

### Fractional atomic coordinates and isotropic or equivalent isotropic displacement parameters (Å^2^)

**Table:**

	*x*	*y*	*z*	*U*_iso_*/*U*_eq_	Occ. (<1)
O1	0.3004 (4)	0.22049 (16)	0.28302 (10)	0.0597 (7)	
O2	−0.4436 (4)	0.45678 (17)	0.41002 (9)	0.0615 (8)	
N1	0.4194 (5)	0.3569 (2)	0.26015 (14)	0.0762 (11)	
N2	0.2304 (5)	0.3662 (2)	0.29375 (13)	0.0731 (11)	
N3	0.9831 (6)	0.1428 (3)	0.17039 (15)	0.0904 (12)	
N4	−0.6818 (5)	0.3527 (2)	0.43895 (13)	0.0726 (10)	
N5	−0.7648 (5)	0.4388 (2)	0.45273 (14)	0.0734 (11)	
N6	−0.6473 (7)	0.7838 (2)	0.45477 (15)	0.0903 (12)	
C1	−0.2506 (6)	0.1407 (3)	0.37961 (15)	0.0586 (11)	
C2	−0.0691 (6)	0.1670 (2)	0.34870 (15)	0.0587 (11)	
H2	0.0240	0.1228	0.3342	0.070*	
C3	−0.0221 (6)	0.2581 (2)	0.33868 (15)	0.0541 (10)	
C4	−0.1606 (6)	0.3249 (3)	0.36019 (14)	0.0584 (11)	
H4	−0.1300	0.3858	0.3540	0.070*	
C5	−0.3437 (6)	0.3001 (2)	0.39072 (14)	0.0537 (11)	
C6	−0.3841 (6)	0.2088 (3)	0.40024 (15)	0.0620 (11)	
H6	−0.5062	0.1929	0.4214	0.074*	
C7	0.1671 (6)	0.2853 (3)	0.30558 (15)	0.0588 (11)	
C8	0.4546 (7)	0.2716 (3)	0.25547 (16)	0.0592 (11)	
C9	0.6312 (6)	0.2249 (3)	0.22503 (15)	0.0587 (11)	
C10	0.6450 (7)	0.1324 (3)	0.22164 (16)	0.0752 (13)	
H10	0.5367	0.0957	0.2375	0.090*	
C11	0.8243 (8)	0.0949 (3)	0.19406 (18)	0.0893 (16)	
H11	0.8334	0.0321	0.1921	0.107*	
C12	0.9652 (7)	0.2322 (4)	0.17330 (18)	0.0845 (15)	
H12	1.0737	0.2672	0.1563	0.101*	
C13	0.7938 (7)	0.2763 (3)	0.20023 (15)	0.0716 (13)	
H13	0.7887	0.3392	0.2015	0.086*	
C14	−0.4945 (7)	0.3669 (3)	0.41435 (15)	0.0587 (11)	
C15	−0.6198 (6)	0.4971 (3)	0.43581 (14)	0.0571 (11)	
C16	−0.6269 (6)	0.5958 (3)	0.44115 (14)	0.0577 (11)	
C17	−0.8051 (6)	0.6360 (3)	0.46777 (15)	0.0709 (13)	
H17	−0.9210	0.6009	0.4819	0.085*	
C18	−0.8076 (7)	0.7293 (3)	0.47298 (17)	0.0782 (14)	
H18	−0.9297	0.7554	0.4904	0.094*	
C19	−0.4754 (8)	0.7431 (3)	0.42957 (19)	0.0953 (16)	
H19	−0.3601	0.7796	0.4164	0.114*	
C20	−0.4581 (7)	0.6506 (3)	0.42176 (16)	0.0746 (13)	
H20	−0.3352	0.6261	0.4038	0.090*	
C21	−0.2996 (7)	0.0404 (3)	0.3923 (2)	0.0689 (12)	
C22	−0.2044 (17)	0.0225 (5)	0.4471 (3)	0.104 (3)	0.781 (13)
H22A	−0.2842	0.0588	0.4724	0.156*	0.781 (13)
H22B	−0.0457	0.0377	0.4482	0.156*	0.781 (13)
H22C	−0.2231	−0.0404	0.4557	0.156*	0.781 (13)
C23	−0.5473 (10)	0.0201 (4)	0.3857 (5)	0.138 (6)	0.781 (13)
H23A	−0.5737	−0.0429	0.3925	0.207*	0.781 (13)
H23B	−0.5946	0.0342	0.3502	0.207*	0.781 (13)
H23C	−0.6329	0.0561	0.4100	0.207*	0.781 (13)
C24	−0.1655 (15)	−0.0238 (4)	0.3541 (3)	0.092 (3)	0.781 (13)
H24A	−0.2110	−0.0854	0.3600	0.139*	0.781 (13)
H24B	−0.0051	−0.0179	0.3609	0.139*	0.781 (13)
H24C	−0.1984	−0.0074	0.3181	0.139*	0.781 (13)
C25	−0.393 (5)	−0.0078 (13)	0.3436 (7)	0.080 (11)	0.219 (13)
H25A	−0.5298	−0.0398	0.3526	0.121*	0.219 (13)
H25B	−0.2824	−0.0500	0.3309	0.121*	0.219 (13)
H25C	−0.4270	0.0358	0.3165	0.121*	0.219 (13)
C26	−0.116 (6)	−0.0169 (19)	0.419 (2)	0.20 (3)	0.219 (13)
H26A	−0.1786	−0.0467	0.4492	0.303*	0.219 (13)
H26B	0.0077	0.0212	0.4298	0.303*	0.219 (13)
H26C	−0.0624	−0.0614	0.3943	0.303*	0.219 (13)
C27	−0.483 (5)	0.0375 (17)	0.4371 (11)	0.125 (15)	0.219 (13)
H27A	−0.6150	0.0714	0.4261	0.187*	0.219 (13)
H27B	−0.4211	0.0636	0.4689	0.187*	0.219 (13)
H27C	−0.5261	−0.0242	0.4437	0.187*	0.219 (13)

### Atomic displacement parameters (Å^2^)

**Table:**

	*U*^11^	*U*^22^	*U*^33^	*U*^12^	*U*^13^	*U*^23^
O1	0.0732 (17)	0.0460 (16)	0.0602 (18)	0.0062 (14)	0.0133 (14)	0.0036 (14)
O2	0.0596 (17)	0.0577 (18)	0.0676 (19)	−0.0027 (14)	0.0171 (14)	−0.0041 (15)
N1	0.086 (3)	0.051 (2)	0.092 (3)	0.002 (2)	0.025 (2)	0.013 (2)
N2	0.077 (2)	0.051 (2)	0.092 (3)	0.0021 (19)	0.030 (2)	0.010 (2)
N3	0.099 (3)	0.086 (3)	0.087 (3)	0.008 (3)	0.022 (2)	−0.001 (3)
N4	0.069 (2)	0.064 (2)	0.085 (3)	−0.0093 (19)	0.021 (2)	−0.009 (2)
N5	0.066 (2)	0.066 (2)	0.089 (3)	−0.008 (2)	0.027 (2)	−0.006 (2)
N6	0.119 (3)	0.067 (3)	0.086 (3)	0.000 (2)	0.030 (2)	−0.004 (2)
C1	0.061 (3)	0.052 (3)	0.063 (3)	−0.004 (2)	−0.001 (2)	0.000 (2)
C2	0.062 (3)	0.048 (3)	0.065 (3)	0.0057 (19)	0.000 (2)	−0.007 (2)
C3	0.059 (3)	0.045 (2)	0.058 (3)	0.003 (2)	0.005 (2)	−0.001 (2)
C4	0.066 (3)	0.052 (3)	0.057 (3)	−0.006 (2)	−0.001 (2)	0.002 (2)
C5	0.062 (3)	0.047 (3)	0.052 (3)	−0.004 (2)	0.006 (2)	−0.005 (2)
C6	0.060 (3)	0.068 (3)	0.059 (3)	−0.014 (2)	0.007 (2)	0.005 (2)
C7	0.066 (3)	0.050 (3)	0.060 (3)	0.002 (2)	0.009 (2)	0.007 (2)
C8	0.069 (3)	0.054 (3)	0.055 (3)	−0.006 (2)	0.011 (2)	0.019 (2)
C9	0.073 (3)	0.058 (3)	0.046 (3)	0.002 (2)	0.009 (2)	0.005 (2)
C10	0.099 (3)	0.059 (3)	0.068 (3)	0.002 (3)	0.023 (3)	0.001 (3)
C11	0.116 (4)	0.066 (3)	0.086 (4)	0.015 (3)	0.026 (3)	−0.001 (3)
C12	0.087 (4)	0.081 (4)	0.086 (4)	−0.002 (3)	0.026 (3)	0.009 (3)
C13	0.086 (3)	0.054 (3)	0.075 (3)	0.001 (2)	0.015 (3)	0.009 (2)
C14	0.065 (3)	0.050 (3)	0.062 (3)	−0.008 (2)	0.006 (2)	−0.003 (2)
C15	0.053 (3)	0.063 (3)	0.056 (3)	−0.002 (2)	0.014 (2)	−0.006 (2)
C16	0.063 (3)	0.065 (3)	0.045 (3)	−0.001 (2)	0.011 (2)	−0.002 (2)
C17	0.069 (3)	0.072 (3)	0.073 (3)	−0.004 (2)	0.019 (2)	−0.007 (3)
C18	0.086 (3)	0.068 (3)	0.081 (3)	0.013 (3)	0.019 (3)	−0.007 (3)
C19	0.116 (4)	0.070 (4)	0.101 (4)	−0.008 (3)	0.040 (3)	0.005 (3)
C20	0.078 (3)	0.068 (3)	0.079 (3)	−0.002 (3)	0.028 (2)	0.001 (3)
C21	0.077 (3)	0.045 (3)	0.086 (4)	0.002 (2)	0.011 (3)	0.010 (3)
C22	0.154 (9)	0.051 (5)	0.107 (7)	0.007 (5)	−0.004 (6)	0.027 (5)
C23	0.056 (4)	0.052 (5)	0.305 (19)	−0.018 (3)	−0.024 (7)	0.032 (8)
C24	0.123 (8)	0.046 (4)	0.108 (6)	−0.020 (4)	0.003 (6)	−0.009 (4)
C25	0.13 (3)	0.044 (13)	0.065 (16)	−0.022 (16)	−0.008 (16)	0.002 (11)
C26	0.22 (4)	0.06 (2)	0.32 (8)	0.04 (2)	−0.17 (5)	−0.01 (3)
C27	0.21 (4)	0.09 (2)	0.07 (2)	−0.05 (2)	0.02 (2)	−0.013 (17)

### Geometric parameters (Å, °)

**Table:**

O1—C7	1.366 (4)	C13—H13	0.9300
O1—C8	1.375 (4)	C15—C16	1.465 (5)
O2—C15	1.367 (4)	C16—C17	1.385 (4)
O2—C14	1.365 (4)	C16—C20	1.375 (5)
N1—C8	1.282 (4)	C17—C18	1.384 (5)
N1—N2	1.413 (4)	C17—H17	0.9300
N2—C7	1.287 (4)	C18—H18	0.9300
N3—C11	1.320 (5)	C19—C20	1.384 (5)
N3—C12	1.326 (5)	C19—H19	0.9300
N4—C14	1.288 (4)	C20—H20	0.9300
N4—N5	1.407 (4)	C21—C22	1.513 (7)
N5—C15	1.288 (4)	C21—C23	1.494 (6)
N6—C18	1.326 (4)	C21—C24	1.573 (6)
N6—C19	1.343 (5)	C21—C25	1.523 (13)
C1—C2	1.385 (5)	C21—C26	1.521 (14)
C1—C6	1.382 (5)	C21—C27	1.575 (11)
C1—C21	1.542 (5)	C22—H22A	0.9600
C2—C3	1.396 (4)	C22—H22B	0.9600
C2—H2	0.9300	C22—H22C	0.9600
C3—C4	1.394 (4)	C23—H23A	0.9600
C3—C7	1.456 (5)	C23—H23B	0.9600
C4—C5	1.381 (4)	C23—H23C	0.9600
C4—H4	0.9300	C24—H24A	0.9600
C5—C6	1.390 (4)	C24—H24B	0.9600
C5—C14	1.458 (4)	C24—H24C	0.9600
C6—H6	0.9300	C25—H25A	0.9600
C8—C9	1.471 (5)	C25—H25B	0.9600
C9—C13	1.377 (4)	C25—H25C	0.9600
C9—C10	1.371 (5)	C26—H26A	0.9600
C10—C11	1.386 (5)	C26—H26B	0.9600
C10—H10	0.9300	C26—H26C	0.9600
C11—H11	0.9300	C27—H27A	0.9600
C12—C13	1.384 (5)	C27—H27B	0.9600
C12—H12	0.9300	C27—H27C	0.9600
			
C7—O1—C8	102.2 (3)	N5—C15—C16	128.0 (4)
C15—O2—C14	102.5 (3)	O2—C15—C16	120.0 (3)
C8—N1—N2	106.3 (3)	C17—C16—C20	118.3 (4)
C7—N2—N1	106.3 (3)	C17—C16—C15	119.6 (4)
C11—N3—C12	116.8 (4)	C20—C16—C15	122.1 (4)
C14—N4—N5	105.9 (3)	C16—C17—C18	118.8 (4)
C15—N5—N4	106.8 (3)	C16—C17—H17	120.6
C18—N6—C19	115.7 (4)	C18—C17—H17	120.6
C2—C1—C6	116.9 (4)	N6—C18—C17	124.3 (4)
C2—C1—C21	122.3 (4)	N6—C18—H18	117.9
C6—C1—C21	120.8 (3)	C17—C18—H18	117.9
C1—C2—C3	121.9 (3)	N6—C19—C20	124.5 (4)
C1—C2—H2	119.0	N6—C19—H19	117.7
C3—C2—H2	119.0	C20—C19—H19	117.7
C4—C3—C2	119.5 (3)	C16—C20—C19	118.4 (4)
C4—C3—C7	118.8 (3)	C16—C20—H20	120.8
C2—C3—C7	121.7 (3)	C19—C20—H20	120.8
C5—C4—C3	119.5 (4)	C23—C21—C22	114.7 (6)
C5—C4—H4	120.2	C23—C21—C1	110.7 (4)
C3—C4—H4	120.2	C22—C21—C1	106.9 (4)
C4—C5—C6	119.3 (3)	C23—C21—C24	107.7 (6)
C4—C5—C14	122.0 (4)	C22—C21—C24	106.0 (5)
C6—C5—C14	118.6 (3)	C1—C21—C24	110.8 (4)
C5—C6—C1	122.8 (3)	C21—C22—H22A	109.4
C5—C6—H6	118.6	C21—C22—H22B	109.5
C1—C6—H6	118.6	C21—C22—H22C	109.5
N2—C7—O1	112.6 (3)	C21—C23—H23A	109.5
N2—C7—C3	127.9 (4)	C21—C23—H23B	109.4
O1—C7—C3	119.4 (3)	C21—C23—H23C	109.5
N1—C8—O1	112.6 (3)	C21—C24—H24A	109.5
N1—C8—C9	128.7 (3)	C21—C24—H24B	109.5
O1—C8—C9	118.7 (3)	C21—C24—H24C	109.5
C13—C9—C10	118.6 (4)	C21—C25—H25A	109.6
C13—C9—C8	118.5 (4)	C21—C25—H25B	109.4
C10—C9—C8	122.9 (4)	H25A—C25—H25B	109.5
C9—C10—C11	118.4 (4)	C21—C25—H25C	109.5
C9—C10—H10	120.8	H25A—C25—H25C	109.5
C11—C10—H10	120.8	H25B—C25—H25C	109.5
N3—C11—C10	124.0 (4)	C21—C26—H26A	109.3
N3—C11—H11	118.0	C21—C26—H26B	109.5
C10—C11—H11	118.0	H26A—C26—H26B	109.5
N3—C12—C13	123.6 (4)	C21—C26—H26C	109.6
N3—C12—H12	118.2	H26A—C26—H26C	109.5
C13—C12—H12	118.2	H26B—C26—H26C	109.5
C9—C13—C12	118.5 (4)	C21—C27—H27A	109.4
C9—C13—H13	120.7	C21—C27—H27B	109.6
C12—C13—H13	120.7	H27A—C27—H27B	109.5
N4—C14—O2	112.7 (3)	C21—C27—H27C	109.5
N4—C14—C5	127.9 (4)	H27A—C27—H27C	109.5
O2—C14—C5	119.3 (3)	H27B—C27—H27C	109.5
N5—C15—O2	112.0 (4)		
			
C8—N1—N2—C7	−0.9 (5)	C11—N3—C12—C13	1.2 (8)
C14—N4—N5—C15	−0.7 (4)	C10—C9—C13—C12	−0.5 (6)
C6—C1—C2—C3	0.0 (6)	C8—C9—C13—C12	177.5 (4)
C21—C1—C2—C3	−178.1 (4)	N3—C12—C13—C9	−0.7 (7)
C1—C2—C3—C4	0.0 (6)	N5—N4—C14—O2	−0.3 (5)
C1—C2—C3—C7	−179.2 (4)	N5—N4—C14—C5	−178.5 (4)
C2—C3—C4—C5	−0.6 (6)	C15—O2—C14—N4	1.1 (4)
C7—C3—C4—C5	178.6 (4)	C15—O2—C14—C5	179.4 (3)
C3—C4—C5—C6	1.2 (6)	C4—C5—C14—N4	172.5 (4)
C3—C4—C5—C14	179.9 (4)	C6—C5—C14—N4	−8.7 (6)
C4—C5—C6—C1	−1.2 (6)	C4—C5—C14—O2	−5.5 (6)
C14—C5—C6—C1	−180.0 (4)	C6—C5—C14—O2	173.2 (3)
C2—C1—C6—C5	0.6 (6)	N4—N5—C15—O2	1.4 (5)
C21—C1—C6—C5	178.7 (4)	N4—N5—C15—C16	−178.9 (4)
N1—N2—C7—O1	0.7 (5)	C14—O2—C15—N5	−1.5 (4)
N1—N2—C7—C3	−179.2 (4)	C14—O2—C15—C16	178.7 (4)
C8—O1—C7—N2	−0.2 (4)	N5—C15—C16—C17	0.9 (7)
C8—O1—C7—C3	179.7 (4)	O2—C15—C16—C17	−179.4 (3)
C4—C3—C7—N2	1.7 (7)	N5—C15—C16—C20	179.0 (4)
C2—C3—C7—N2	−179.1 (4)	O2—C15—C16—C20	−1.3 (6)
C4—C3—C7—O1	−178.2 (3)	C20—C16—C17—C18	1.0 (6)
C2—C3—C7—O1	1.0 (6)	C15—C16—C17—C18	179.2 (4)
N2—N1—C8—O1	0.9 (5)	C19—N6—C18—C17	0.5 (7)
N2—N1—C8—C9	−179.8 (4)	C16—C17—C18—N6	−1.1 (7)
C7—O1—C8—N1	−0.5 (5)	C18—N6—C19—C20	0.2 (8)
C7—O1—C8—C9	−179.9 (3)	C17—C16—C20—C19	−0.4 (6)
N1—C8—C9—C13	4.7 (7)	C15—C16—C20—C19	−178.5 (4)
O1—C8—C9—C13	−176.0 (4)	N6—C19—C20—C16	−0.3 (8)
N1—C8—C9—C10	−177.4 (5)	C2—C1—C21—C23	−135.0 (7)
O1—C8—C9—C10	1.9 (6)	C6—C1—C21—C23	47.0 (8)
C13—C9—C10—C11	1.0 (7)	C2—C1—C21—C22	99.4 (6)
C8—C9—C10—C11	−176.9 (4)	C6—C1—C21—C22	−78.6 (6)
C12—N3—C11—C10	−0.7 (7)	C2—C1—C21—C24	−15.6 (7)
C9—C10—C11—N3	−0.4 (7)	C6—C1—C21—C24	166.4 (5)

### Hydrogen-bond geometry (Å, °)

**Table:**

*D*—H···*A*	*D*—H	H···*A*	*D*···*A*	*D*—H···*A*
C2—H2···O1	0.93	2.54	2.860 (4)	101
C6—H6···N4	0.93	2.61	2.928 (5)	100
C11—H11···N2^i^	0.93	2.50	3.406 (6)	164
C17—H17···N5^ii^	0.93	2.56	3.430 (5)	156

Symmetry codes: (i) −*x*+1, *y*−1/2, −*z*+1/2; (ii) −*x*−2, −*y*+1, −*z*+1.
